# Multi-omics reveals largely distinct transcript- and protein-level responses to the environment in an intertidal mussel

**DOI:** 10.1242/jeb.245962

**Published:** 2023-11-21

**Authors:** Lani U. Gleason, Florian J. Fekete, Richelle L. Tanner, W. Wesley Dowd

**Affiliations:** ^1^ Department of Biological Sciences, California State University Sacramento, Sacramento, CA 95819, USA; ^2^ School of Biological Sciences, Washington State University, Pullman, WA 99163, USA

**Keywords:** RNA-seq, Proteomics, Mollusk, Rocky intertidal, Environmental stress, *Mytilus californianus*

## Abstract

Organismal responses to stressful environments are influenced by numerous transcript- and protein-level mechanisms, and the relationships between expression changes at these levels are not always straightforward. Here, we used paired transcriptomic and proteomic datasets from two previous studies from gill of the California mussel, *Mytilus californianus*, to explore how simultaneous transcript and protein abundance patterns may diverge under different environmental scenarios*.* Field-acclimatized mussels were sampled from two disparate intertidal sites; individuals from one site were subjected to three further treatments (common garden, low-intertidal or high-intertidal outplant) that vary in temperature and feeding time. Assessing 1519 genes shared between the two datasets revealed that both transcript and protein expression patterns differentiated the treatments at a global level, despite numerous underlying discrepancies. There were far more instances of differential expression between treatments in transcript only (1451) or protein only (226) than of the two levels shifting expression concordantly (68 instances). Upregulated expression of cilium-associated transcripts (likely related to feeding) was associated with relatively benign field treatments. In the most stressful treatment, transcripts, but not proteins, for several molecular chaperones (including heat shock proteins and endoplasmic reticulum chaperones) were more abundant, consistent with a threshold model for induction of translation of constitutively available mRNAs. Overall, these results suggest that the relative importance of transcript- and protein-level regulation (translation and/or turnover) differs among cellular functions and across specific microhabitats or environmental contexts. Furthermore, the degree of concordance between transcript and protein expression can vary across benign versus acutely stressful environmental conditions.

## Teaser

**Summary:** The importance of transcript- and protein-level regulation differs across cellular functions and environmental contexts, and transcript and protein expression have higher concordance in stressful conditions.

## INTRODUCTION

Plasticity in the molecular phenotype, such as changes in transcript and protein abundance, can facilitate tolerance to climate change-induced environmental changes (Rivera et al., 2021; Seebacher et al., 2015; Visser, 2008). Yet, more information is needed about plastic expression responses to ecologically relevant conditions to better understand how organisms will fare in rapidly changing habitats. The relationship between different components of the molecular phenotype also warrants further investigation. For example, proteins best represent the molecular phenotype because protein function more directly affects fitness than mRNA abundance (Feder and Walser, 2005; Kültz, 2015). Translation elongation (Nie et al., 2006), translation regulation (Brockmann et al., 2007), tRNA abundance (Torrent et al., 2018), ribosomal density, and mRNA and protein stability (Tuller et al., 2007) affect whether a change in transcript abundance results in a corresponding change in protein abundance (Gunawardana and Niranjan, 2013; Vogel and Marcotte, 2012; see Wang et al., 2014 for a summary). In fact, these factors generally result in a relatively modest correlation between transcript and protein expression under both steady-state (de Sousa Abreu et al., 2009; Schwanhäusser et al., 2011) and changing conditions (e.g. Gan et al., 2013; Lan et al., 2012). Therefore, complementary use of RNA-seq and proteomics techniques is essential for fully understanding gene expression (Buccitelli and Selbach, 2020). Moreover, even with the inclusion of proteomic data, connections to measurable physiological phenotypes relevant to survival in stressful environments, such as enzyme activity, are not always apparent. These relationships are further complicated by the differing time scales of transcript expression and protein translation, as well as the multi-faceted patterns of variation in natural systems (Gedeon and Bokes, 2012). For instance, many transcriptome-wide assessments are conducted in the lab and focus on a single environmental stressor (e.g. Chen et al., 2022; Gleason and Burton, 2015; Lee et al., 2021; Moreira et al., 2021; Zhou et al., 2021; Zhu et al., 2022). Such studies cannot be used to assess how organisms will respond to the same stressor in the field, because variation in other factors, such as the occurrence of simultaneous multiple stressors, can cause synergistic gene expression responses (DeBiasse and Kelly, 2016; Gleason, 2019).

Sessile intertidal organisms provide a useful model for examining the molecular bases of plasticity in the field. A variety of intertidal organisms already live close to their upper thermal limit and are thus susceptible to climate change (Dong et al., 2022; Somero, 2010). Sessile invertebrates can be especially vulnerable because they cannot migrate to more suitable habitats as adults; instead, movement occurs during the planktonic larval phase. Longer planktonic larval durations facilitate high gene flow that can homogenize the gene pool and impede local adaptation to unique environmental conditions (Parsons, 1998; Struhsaker, 1968; Yamada, 1989). Indeed, in marine invertebrates with higher dispersal, plasticity is more common than local adaptation (Hollander, 2008; Kawecki and Ebert, 2004; Warner, 1997; but see Sasaki et al., 2022). The intertidal California mussel (*Mytilus californianus*) is one such sessile invertebrate with a long planktonic larval duration, high gene flow and relatively high genetic homogeneity across its range (Addison et al., 2008; López-Duarte et al., 2012). Post-settlement *M. californianus* face multiple stressors in the rocky intertidal zone, including limited feeding time and high temperatures. *Mytilus californianus* is vulnerable to rising temperatures; several recent heat waves along the western coast of the USA have caused mass mortality of the species (Brownlee, 2022; Harley, 2008; Mislan and Wethey, 2015). Furthermore, *M. californianus* can only filter-feed during high tide periods (Winter, 1978). Thus, feeding time varies over very short distances, with mussels at higher intertidal sites having the least amount of time available to feed (Dehnel, 1956; Dowd et al., 2013; Miller and Dowd, 2017, 2019). This variation in food availability across microsites affects ATP-generating and antioxidant enzyme capacities (Dowd et al., 2013) and growth (Connor and Robles, 2015) of mussels. Because *M. californianus* provides food and shelter for a wide variety of other intertidal organisms, mortality events for this species could drastically alter the intertidal ecosystem (Suchanek, 1980, 1992; Harley, 2008; Smith et al., 2006).

In the rocky intertidal zone, both spatial and temporal variation affect inhabitants. Within *M. californianus* beds (Morris et al., 1980), temperature differences across fine microhabitat scales can meet or even exceed average latitudinal temperature differences across the species' range from Baja California to Alaska (Denny et al., 2011). This spatial variation can result in significantly different amounts of oxidative DNA damage and osmolyte levels in individuals only centimeters apart (Gleason et al., 2017). Temporal variation in the intertidal also exists as a function of the tidal cycle. Low tides, especially during warm daytime periods (Helmuth et al., 2006), result in intermittent air exposure and emersion-associated stressors that affect physiology, distribution and, ultimately, survival of intertidal organisms (Connell, 1972; Foster, 1986; Solan and Whiteley, 2016). It is well documented that these persistent fluctuations create a physiologically challenging environment (Tomanek and Helmuth, 2002), and *M. californianus* mussels from unique intertidal microhabitats exhibit physiological plasticity in metabolic rate and antioxidant capacities when exposed to different environmental contexts that vary in the frequency and intensity of stressful events (Jimenez et al., 2015). However, we do not fully understand the molecular mechanisms underlying tolerance to this extreme spatial and temporal variation, nor is there sufficient understanding of how plasticity modulates these molecular mechanisms.

To cope with intermittent periods of environmental stress, intertidal organisms might induce expression of genes when required by the conditions or constitutively express genes involved in the stress response. In some cases, intertidal mollusks, including *M. californianus*, use a strategy of constitutive expression (Clark et al., 2018; Dong et al., 2008; Gleason and Burton, 2015; Gracey et al., 2008). For instance, *M. californianus* individuals show high transcript expression of molecular chaperones involved in unfolded protein binding even during non-stressful high tide conditions (Gracey et al., 2008). Although patterns of transcriptional regulation are emerging from a number of RNA-sequencing studies (e.g. Barshis et al., 2013; Chen et al., 2022; Gleason and Burton, 2015; Lee et al., 2021; Moreira et al., 2021; Schoville et al., 2012; Zhou et al., 2021; Zhu et al., 2022), the relationship between transcript levels and post-transcriptional processes that result in varying levels of functional proteins in the intertidal zone remains largely unknown.

This objective of this study was to investigate the effects of acclimatization to distinct intertidal habitats on transcript and protein expression in *M. californianus* individuals. We exposed adult *M. californianus* to various field and lab treatments that vary in degrees of environmental stress and then performed both RNA-seq and proteomics analyses to better understand molecular phenotype plasticity. Specifically, we use the paired transcriptome and proteome datasets to address the following questions: (1) can global transcriptome and/or proteome profiles differentiate ecologically relevant treatment conditions; (2) are treatment differences in the transcriptome consistently reflected in the proteome, or is protein expression more tightly coupled to transcript expression for particular cellular functions or in certain conditions; and (3) which components of the transcriptome and/or proteome are most correlated with physiological measurements on the same tissue?

## MATERIALS AND METHODS

### Experimental design

The treatments and individual mussel samples for this study are the same ‘baseline’ samples as those described in detail in Jimenez et al. (2015) (gill tissue physiological measurements) and in Tanner et al. (2022) (gill tissue inter-individual variation in transcript and protein levels). Here, we synthesize data from those two studies.

**Fig. 1.**
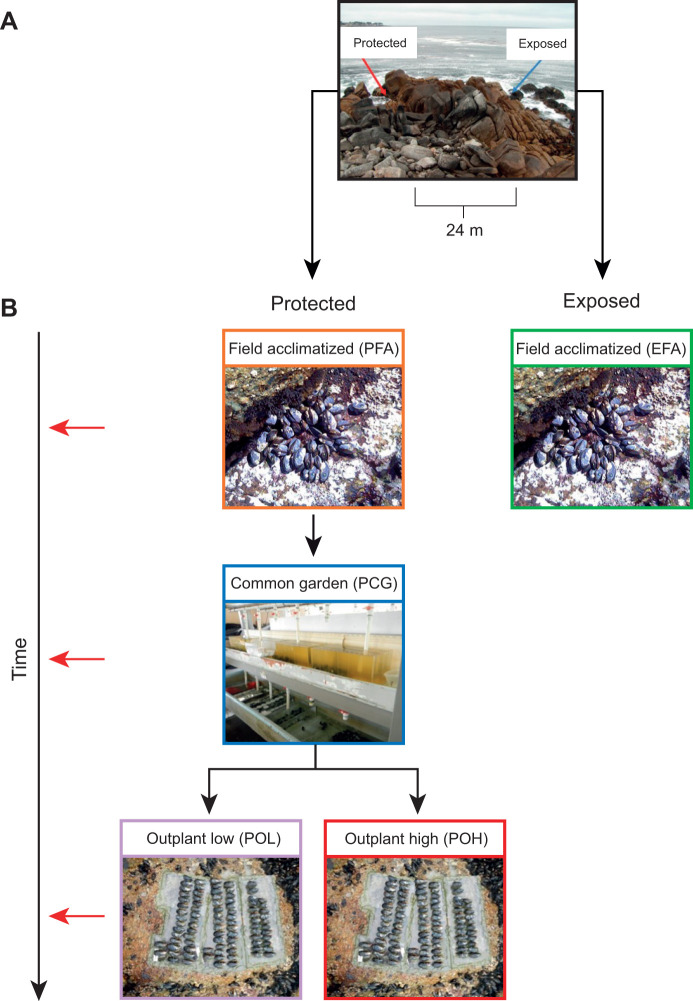
**Experimental design showing origin sites and sampling times for each treatment group.** (A) Experiments were conducted at Hopkins Marine Station, Pacific Grove, CA, USA, using mussels from two different origin sites that differ in wave exposure (wave-exposed, blue arrow, cool; wave-protected, red arrow, warm). (B) Mussels were designated into one of five treatment groups and sampled at three different time points, as indicated by the horizontal red arrows on the left ‘time’ axis. Mussels from the exposed site were sampled directly from the field (EFA). Mussels from the protected site were sampled directly from the field (PFA) and also after exposure to 28 days of laboratory common garden conditions (PCG). Additional mussels were taken from the common garden and outplanted back into the field for 28 days at two different heights on the shore: a low, relatively cool site (POL), and a high, relatively warm site (POH). Colored borders for each of the five treatment groups in B match color-coding used in Figs 2 and S2. This figure is modified from Fig. S1 in Tanner et al. (2022).

Adult *Mytilus californianus* Conrad 1837 mussels (60–70 mm in shell height) were sampled from five field and lab treatments in 2014 (Fig. 1). These treatments represent environmental contexts that vary in the mean daily maximum body temperature and available feeding time (Tanner et al., 2022; Table 1). The treatments and sampling time points were chosen to assess cumulative effects of environmental history manipulations on the molecular phenotype, rather than the response to an acute stressor. Briefly, two groups of mussels were first collected from a cool wave-exposed site (*n=*9) and a warm wave-protected site (*n=*9) at Hopkins Marine Station in Pacific Grove, CA, USA (36.6203°N, 121.9042°W). These field-acclimatized mussels were compared between the two field sites. For this study, only the three subsequent treatments pertaining to mussels collected from the wave-protected site were used, because their physiological differences across treatments were more robust (Jimenez et al., 2015). Mussels from the protected site were brought into a common garden lab setting for 1 month (*n=*5) and kept constantly submerged in 13.5±1°C seawater supplied from Monterey Bay. Mussels were fed daily with a commercial bivalve diet (Shellfish Diet 1800; Reed Mariculture) to supplement the natural food in the seawater. Some individuals were sampled at the end of this common garden period, and two other treatment groups were placed back into the field (i.e. they were outplanted) at either a cool low intertidal site (*n=*8) or a hot high intertidal site (*n=*10) for an additional month before they were sampled. All individuals were sampled at a comparable point in the tidal cycle, just as tides were receding from the field sites, to avoid confounding factors from rhythmic gene expression (Elowe and Tomanek, 2021; Gracey et al., 2008). Sample sizes for this study and microhabitat characteristics from previous studies (Denny et al., 2011; Miller and Dowd, 2017, 2019) are shown in Table 1. We did not monitor the body temperatures of the mussels at the time of or prior to sampling, but we expect that temperatures in Table 1 represent the temperatures mussels in this study experienced across microhabitats given the consistent differences at the outplant low and outplant high sites observed in prior studies conducted in two separate years (Miller and Dowd, 2017; Miller and Dowd, 2019; Table 1).

**Fig. 2.**
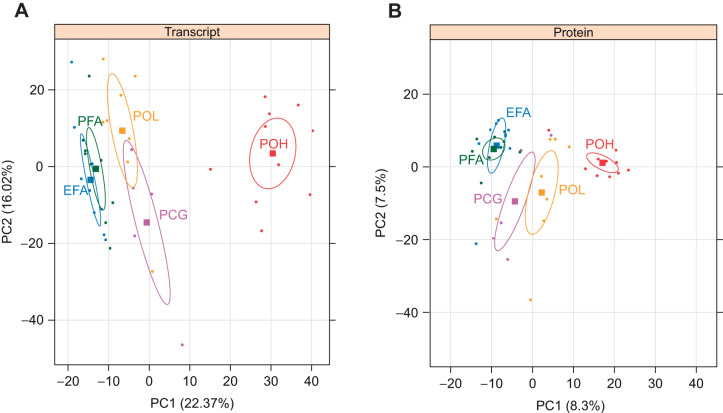
**Principal component analysis (PCA) scores on dimensions 1 (PC1) and 2 (PC2) of expression values for 1519 mussel transcripts and corresponding proteins across five treatments.** (A) Transcripts. (B) Proteins. Ellipses represent 95% confidence intervals for each treatment. Numbers in parentheses on each axis indicate the proportion of variance explained by that PC. Colors represent different treatment groups; each symbol represents one mussel. EFA, exposed field acclimatized, blue, *n*=9; PFA, protected field acclimatized, green, *n*=9; PCG, protected common garden, pink, *n*=5; POL, protected outplant low, yellow, *n*=8; POH, protected outplant high, red, *n*=10.

**Table 1.**
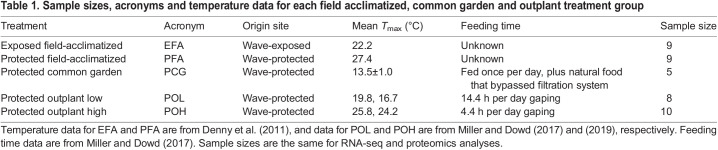
Sample sizes, acronyms and temperature data for each field acclimatized, common garden and outplant treatment group

### RNA sample preparation and sequencing

Mussels were dissected and gill tissue was obtained from each individual, frozen in liquid nitrogen and stored at −80°C. Detailed methods are described in Tanner and Dowd (2019). Briefly, 0.04 g of gill tissue from each individual was ground to a fine powder with a mortar and pestle and RNA was extracted with TRI reagent (Sigma-Aldrich). Total RNA was quantified by UV-Vis spectroscopy (Implen Nanophotometer) and cleaned with the Qiagen RNeasy Mini Plus Kit (Qiagen, MD, USA). Small fragment cDNA library construction, addition of barcodes, and 150 bp paired-end (PE150) and 50 bp paired end (PE50) Illumina HiSeq 2500 rapid-run sequencing were performed by the Genome and Cytometry Core at the University of Southern California. RNA sequence data used for this study are available under BioProject accession no. PRJNA800777 in the NCBI SRA.

### *De novo* transcriptome assembly, annotation and mapping

Sequences were trimmed for ambiguity (a maximum of two ambiguous nucleotides were allowed for PE150 reads and one ambiguous nucleotide was allowed for PE50 reads) and quality (low quality reads below a 0.025 limit were removed) using CLC Genomics Workbench 9.0. For PE150 sequencing, reads shorter than 60 bp were discarded; for PE50 sequencing, reads shorter than 30 bp were discarded. PE150 reads were used to construct a *de novo* transcriptome assembly (Tanner and Dowd, 2019; Tanner et al., 2022). Using the Trinotate v.3.1.1 pipeline, the reference transcriptome containing 130,000 transcripts was annotated using the BLASTnr database. SAMtools v.1.9 and Bowtie v.2.3.2 (Langmead and Salzberg, 2012; Li et al., 2009) were used to map the PE50 reads to the *de novo* transcriptome and TMM normalized expression values were obtained for each transcript of the assembly using Trinotate v.3.1.1. Full details regarding sequencing results and the reference transcriptome assembled can be found in Tanner et al. (2022).

### Protein sample preparation and sequencing

Portions of frozen gill tissue from the same samples described above, resulting in equal sample sizes for RNA-seq and proteomics analyses, were sent to the laboratory of Dietmar Kültz at the University of California, Davis, for proteomics analysis. Detailed methods are described in Tanner and Dowd (2019). Briefly, proteins from membrane, cytosolic and organelle cellular fractions were isolated in liquid nitrogen and digested into peptides using trypsin (Kültz et al., 2013). Protein expression data were obtained through a data-dependent acquisition mode using conventional tandem mass spectrometry (MS1-MS2). The *de novo M. californianus* transcriptome described above was translated to amino acid sequences, and these sequences were used as a reference database for matching proteins to MS/MS spectra. Protein IDs obtained from the bioinformatic search engines Mascot 2.2.7, X!Tandem Alanine, PEAKSC and Byonic 2.12 were considered valid if: (1) matches were based on at least two unique peptides, (2) matches met a protein false-discovery rate (FDR) of 1%, and (3) matches met a peptide FDR of 0.1%. The PCAmethods package in the R Bioconductor suite (Stacklies et al., 2007) was used to impute missing expression values (34.56% of all protein expression values) using the local least squares method. Proteomics data used for this study are available as supplemental data in Tanner and Dowd (2019).

### Identification of contigs with both transcript and protein expression data

After obtaining transcript and protein expression data for each individual, the transcripts and proteins represented in both datasets were identified. Thus, the 130,000 contig reference transcriptome was reduced to a final dataset of 1519 shared transcripts and proteins that was used for subsequent analyses (Tanner et al., 2022). Gene Ontology (GO) (Ashburner et al., 2000) terms were retrieved for each of the annotated 1519 genes/proteins at an *e*-value threshold of 1.0E-6 using Blast2GO.

### Multivariate analyses

To identify multivariate patterns in the RNA-seq and proteomics data, the package FactoMineR (Lê et al., 2008) in R was used to perform principal components analysis (PCA) on the normalized expression values of all samples for (1) RNA-seq and (2) proteomics data. The plotellipses function was used to draw confidence ellipses around each treatment group and to determine whether treatment groups were significantly different from each other. Using the dimdesc function in FactoMineR, the contigs whose expression significantly contributed to the first two principal component dimensions were also determined for each PCA.

### Identification of differentially expressed transcripts and proteins

Five pairwise comparisons were performed to examine plasticity of transcript and protein expression across treatments: (1) EFA versus PFA (origin site effect), (2) PFA versus PCG (response to a benign environment with continuous immersion), (3) PCG versus POL (response to outplanting to a benign intertidal site), (4) PCG versus POH (response to outplanting to a stressful intertidal site) and (5) POL versus POH (differential responses to benign versus stressful intertidal sites). We excluded all comparisons of exposed field acclimatized (EFA) versus protected common garden (PCG), protected outplant low (POL) and protected outplant high (POH) groups, because they involved both a difference in treatment and mussels from different origin sites. We also excluded protected field acclimatized (PFA) versus POL and POH treatments because they were not ‘sequential’ according to the experimental design (e.g. PFA groups were put in a common garden first before outplanting).

RNA-seq read counts and proteomics expression data were separately analyzed using the package edgeR (Robinson et al., 2010) in the statistical environment R (www.CRAN.R-project.org) according to default parameters. Briefly, trimmed mean of M-values (TMM) normalization was performed to eliminate composition biases between treatment group libraries (Robinson and Oshlack, 2010). A negative binomial dispersion was estimated, and differentially expressed transcripts and proteins were identified for each treatment comparison using exact tests (Robinson and Smyth, 2008). The resulting *P*-values were adjusted for multiple hypothesis testing per Benjamini and Hochberg (1995), a relatively conservative method, and genes with an FDR<0.05 were considered to have significantly different expression between treatment groups. Enrichment analyses were performed in Blast2GO to detect any functional GO categories that were overrepresented in the differentially expressed (DE) transcripts and proteins for each comparison (compared with the full set of 1519 genes using Fisher's exact tests).

### Comparing transcript and protein differential expression

After identifying which transcripts and proteins were DE for each treatment pair comparison, Fisher's exact tests were performed to determine whether there was a significant difference between the number of DE transcripts and proteins (i.e. is the transcript expression response to a particular treatment condition significantly larger, or smaller, than the protein expression response?).

In addition to examining which contigs were differentially expressed at the transcript and protein level, results were further examined to determine whether contigs that were DE changed expression levels in the same direction (e.g. higher expression in one treatment group at both the RNA and protein level, or conflicting patterns between RNA and protein). To determine whether genes with certain functions were more commonly found to mirror expression changes at both the RNA and protein level, four different groups of genes were identified: (1) those that are DE in both datasets and share the same direction of expression change; (2) those that are DE at both levels but change expression in opposite directions; (3) those that are DE at the protein but not the transcript level; and (4) those that are DE at the transcript but not the protein level. These four gene lists were generated for each of the five treatment pair comparisons. Functional enrichment analyses were then performed on each of the 20 resulting gene lists.

### Co-expression network analyses

We gained further insight into transcript and protein expression patterns and how they correlate with three physiological measures of antioxidant capacity in gill tissue from the same individuals (Jimenez et al., 2015) using the R package WGCNA (Langfelder and Horvath, 2008). DESeq2 was first used to apply a regularized log transformation on gene and protein expression counts to minimize differences between samples for contigs with small counts and to normalize for library size (Love et al., 2014). After normalization a single consensus network based on both RNA and protein expression of all 1519 contigs was created. However, most protein-specific modules did not have a consensus module counterpart, indicating that the module structures in the RNA and protein expression data were different, and that the consensus modules do not adequately represent the protein data (Fig. S1). Thus, we created separate networks based on only RNA or protein expression of all 1519 contigs across 40 individuals in the five treatments (one PCG individual was excluded owing to missing physiological data) to allow assessment of differences in transcript and protein expression. Connection strengths were obtained from expression similarities using a soft threshold power of 7 and 5 for transcript and protein data, respectively.

Modules of genes that were significantly correlated with mussel gill catalase enzyme activity against hydrogen peroxide [international units (IU) per gram fresh tissue mass (g FM)] and antioxidant activities against peroxyl (µmol Trolox equivalents per gram of tissue) and hydroxyl radicals [arbitrary units (au) per gram of tissue] in RNA expression and protein expression data were identified using the module eigenvalues. Blast2GO enrichment analyses were used to detect any functional GO categories that were overrepresented in these modules that had a significant correlation with physiological metrics. Further intramodular analysis was performed following conservative WGCNA recommendations (https://horvath.genetics.ucla.edu/html/CoexpressionNetwork/Rpackages/WGCNA/Tutorials/) to identify genes most significantly correlated with the trait of interest (gene significance >0.4) that also have high module membership (module membership >0.8). These genes are the most influential, ‘central’ elements of the modules associated with each physiological trait.

## RESULTS

### Expression differences across microhabitats

#### Global patterns

Similar global shifts in both transcript and protein expression across the five field and lab treatments were observed in multivariate space (Fig. 2). The 95% confidence interval ellipses on the transcript expression PCA separated the treatment groups into three distinct clusters along PC1: (1) field-acclimatized exposed and protected, (2) common garden and outplant low, and (3) the stressful outplant high treatment (Fig. 2A). The latter POH cluster showed the most positive loadings on PC1 and was clearly isolated from all other treatments. Genes significantly positively correlated with PC1 (*n*=446) were functionally enriched for unfolded protein binding, protein catabolic processes and response to heat; genes negatively correlated with PC1 (*n*=403) were functionally enriched for cell projections, cilium organization and assembly, and microtubule-based processes (Table S1). Overall, this suggests that the two field-acclimatized treatments differed most from the POH treatment in their overall functional state, with POH being more associated with the stress response and field-acclimatized treatments being more associated with cytoskeleton-related processes (possibly including cell mobility, replication and/or ciliary activity associated with filter feeding). PC2 showed less separation among groups, and genes significantly positively correlated with PC2 were not functionally enriched for any specific GO terms. Genes that were significantly negatively correlated with PC2 included macromolecular biosynthetic processes and structural components of the ribosome related to translation (Table S1).

The protein expression PCA similarly separated the treatments in multivariate space, although the first two PCs explained a lower proportion of the overall variance (Fig. 2B). The 95% confidence interval ellipses differentiated the same three treatment clusters, with POH individuals showing the most positive loadings on PC1 and field-acclimatized individuals showing the most negative loadings on PC1. No GO terms were significantly enriched among proteins correlated with PC1, but several proteins involved in protein binding and folding (10 contigs out of 284 total) showed a significant positive correlation with PC1, and proteins related to structural constituents of the ribosome (11 contigs out of 197 total) were significantly negatively correlated with PC1 (Table S1). There was considerable overlap among treatment groups on PC2. The 348 proteins that were significantly positively correlated with PC2 are functionally enriched for metabolic processes (Table S1).

#### Differential expression

**Fig. 3.**
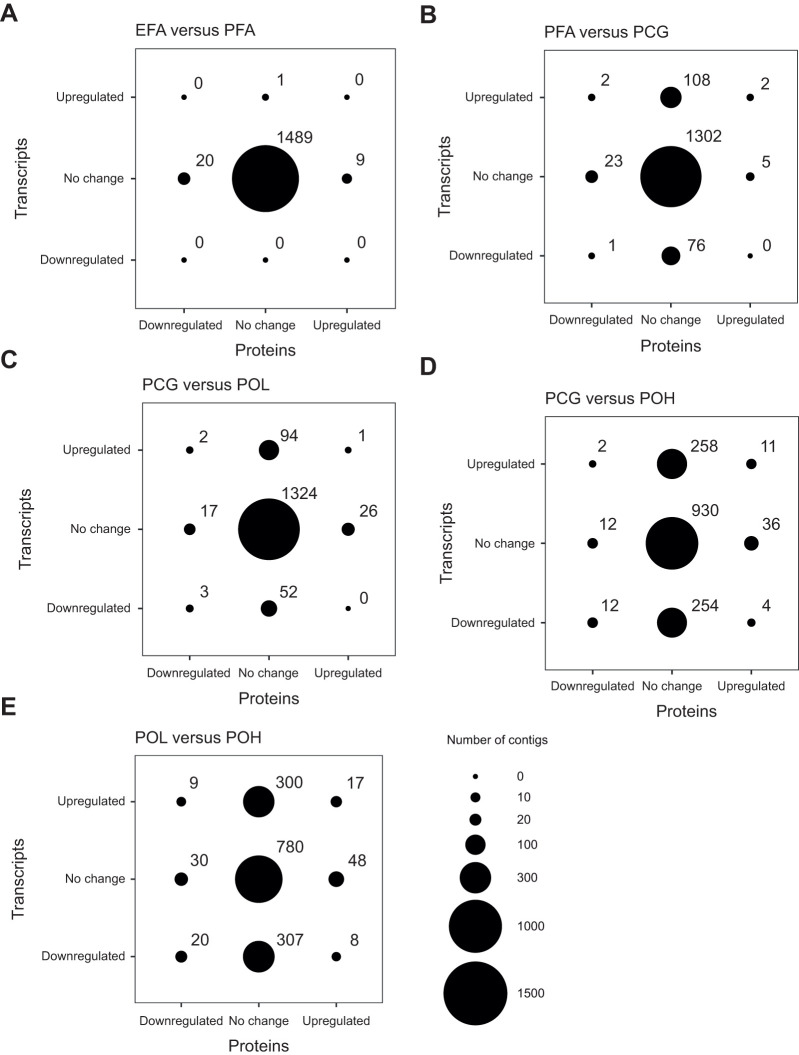
**Bubble charts indicating differential RNA transcript and protein expression for pairwise treatment comparisons.** (A) EFA versus PFA, (B) PFA versus PCG, (C) PCG versus POL, (D) PCG versus POH and (E) POL versus POH. Circle sizes represent the number of genes in each category, with the number of genes listed above each respective circle. The total number of genes for each plot is 1519. The reference group of each comparison is listed first. Upregulated: higher expression in the reference group; downregulated: lower expression in the reference group. Values in the upper right and bottom left of each plot indicate the numbers of gene products upregulated or downregulated at both the transcript and protein level, respectively. EFA, exposed field acclimatized, *n*=9; PFA, protected field acclimatized, *n*=9; PCG, protected common garden, *n*=5; POL, protected outplant low, *n*=8; POH, protected outplant high, *n*=10).

In pair-wise treatment comparisons, 1, 189, 152, 541 and 661 DE transcripts were found for (1) EFA versus PFA, (2) PFA versus PCG, (3) PCG versus POL, (4) PCG versus POH and (5) POL versus POH, respectively (5% FDR correction). The two comparisons including the stressful POH treatment revealed the highest numbers of DE transcripts (Fig. 3D,E). For the field comparison 1, the single DE transcript was unannotated. For comparison 2, no GO terms were significantly overrepresented, but 14 transcripts related to cilium assembly and movement were identified (Table 2). In comparison 3, GO terms related to positive regulation of metabolic processes were significantly overrepresented. These transcripts showed higher expression in the common garden (PCG) than in the field (Table S2). For both outplant high treatment comparisons (PCG versus POH, and POL versus POH), DE transcripts involved in unfolded protein binding and protein folding were overrepresented and showed overall higher expression in the POH group (Table S2). Specifically, for comparison 5, seven of nine DE heat shock proteins (HSPs) were more highly expressed in POH. In addition, four subunits of the T-complex subunit 1, a component of the molecular chaperone tailless complex polypeptide 1 ring complex (TRiC), were also more highly expressed in POH than in POL. Lastly, five transcripts involved in the endoplasmic reticulum (ER) stress pathway and quality control of protein folding in the ER (calnexin, calreticulin, UDP-glucose glycoprotein glycosyltrasferase, ER chaperone BiP, and protein disulfide isomerase A4) were more highly expressed in POH than in POL.

**Table 2.**
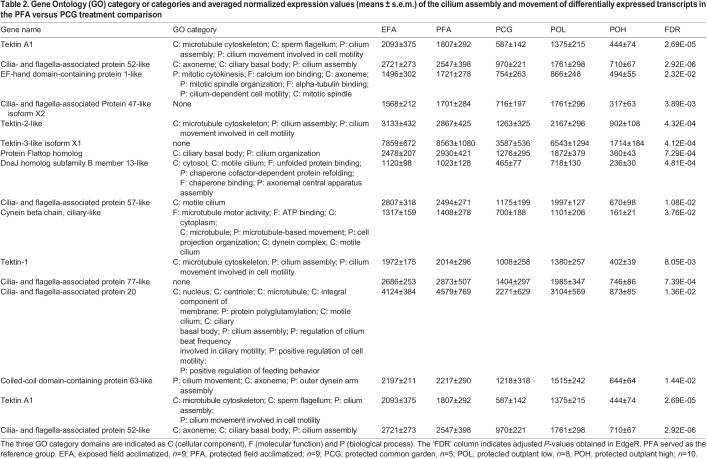
Gene Ontology (GO) category or categories and averaged normalized expression values (means ± s.e.m.) of the cilium assembly and movement of differentially expressed transcripts in the PFA versus PCG treatment comparison

Patterns of DE in the transcriptome were not consistently mirrored in the proteome. Overall, 29, 33, 49, 77 and 132 DE proteins were found in the five treatment comparisons (5% FDR correction). Except for the EFA versus PFA comparison, the number of DE proteins was much lower than the number of DE transcripts. As above for the DE transcripts, the two comparisons including the stressful POH treatment revealed the highest number of DE proteins (Fig. 3D,E). No functional GO terms were found to be overrepresented in any of the treatment comparisons (Table 3). The PCG versus POH comparison identified the highest number of DE proteins relating to the 40S (four contigs) and 60S (one contig) structural constituent of ribosomes; individuals in the PCG group showed higher expression of these ribosomal proteins. Lastly, the POL versus POH comparison showed higher expression of four of six oxidoreductase activity and oxidation–reduction process proteins and three of four microtubule proteins in the POH group. Notably, unlike the transcript comparisons described above, there were no differentially expressed HSPs in the POL versus POH comparison.

**Table 3.**
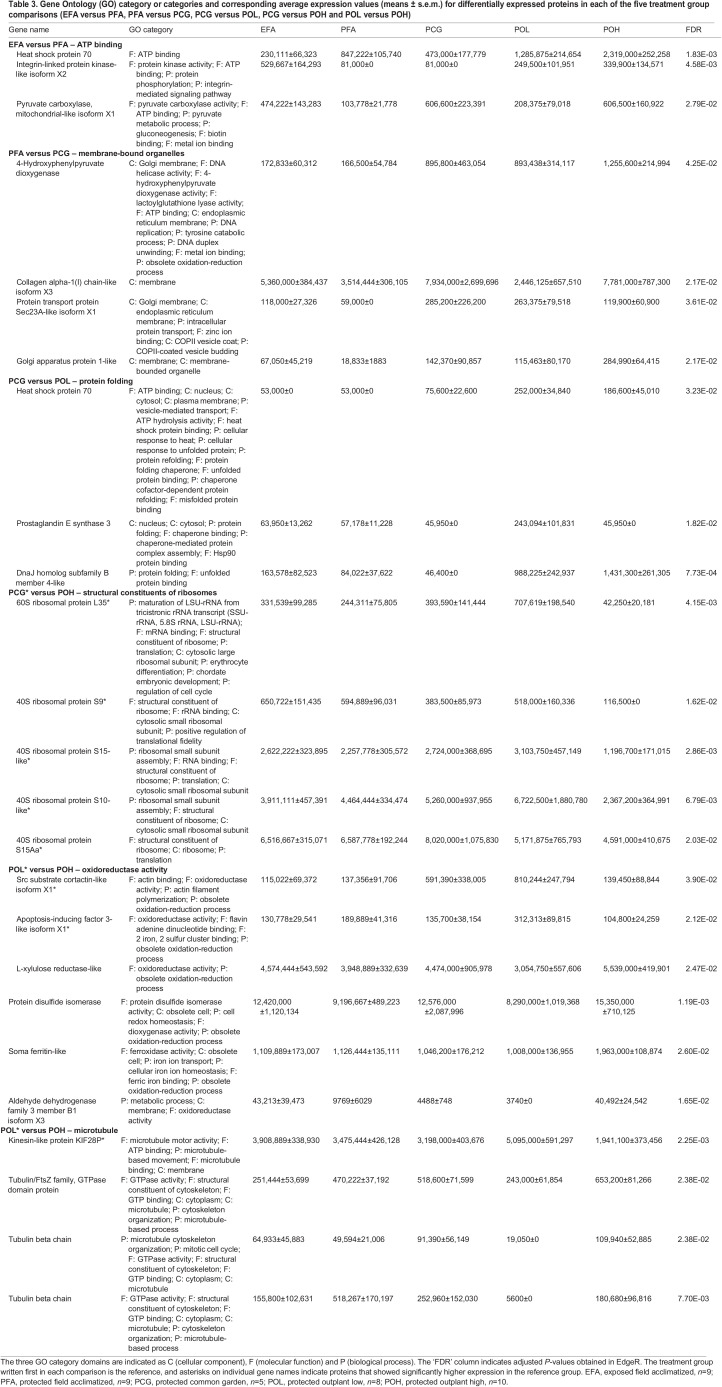
Gene Ontology (GO) category or categories and corresponding average expression values (means ± s.e.m.) for differentially expressed proteins in each of the five treatment group comparisons (EFA versus PFA, PFA versus PCG, PCG versus POL, PCG versus POH and POL versus POH)

#### Comparing transcript and protein differential expression

For each of the five treatment comparisons, the total number of transcripts and proteins whose expression differed was significantly different (Fisher's exact test, *P*<0.0001 in each case).

For each pairwise treatment comparison, we identified four different sets of DE genes: (1) 56 unique genes (68 total instances) were DE at both levels and shared the same direction of expression change, (2) 25 unique genes (28 instances) were DE at both levels but changed expression in opposite directions, (3) 177 unique genes (226 instances) were DE at the protein but not the transcript level, and (4) 853 unique genes (1451 instances) were DE at the transcript but not the protein level (see Table S3 for complete lists).

Only a small percentage of the 1519 genes fell into the first two sets and were DE at both the transcript and the protein level in any treatment comparison; in 71% of those instances [68/(28+68)], the directions of expression changes matched between the two levels (Table S3). Comparisons involving the stressful POH treatment stand out, having both the highest overall numbers and the highest percentages of shared DE genes with the same direction of expression change [23/29 (79%) and 37/54 (69%) for PCG versus POH and POL versus POH, respectively] (Fig. 3D,E). Interestingly, in comparisons involving the POH treatment, genes were almost evenly divided between those consistently upregulated and those consistently downregulated at both levels (Fig. 3D,E). For example, in the POL versus POH comparison, 17 genes were expressed more highly at the transcript and protein level in the POL group; this group included genes such as poly [ADP-ribose] polymerase 3 and apoptosis-inducing factor 3-like isoform X1 (Table S3). In the same comparison, 20 genes were expressed more highly in the POH group, including 78 kDA glucose-regulated protein (an endoplasmic reticulum chaperone), three collagen alpha chains, and other genes with oxidoreductase GO annotations such as protein disulfide isomerase and aldehyde dehydrogenase family 3 member B1 isoform X3. However, no GO terms were overrepresented among the first two gene sets.

A larger group of 177 unique genes fell into the third set and were regulated at the protein but not the transcript level in at least one treatment comparison. Although no GO terms were significantly enriched in any treatment comparison, many genes in this group are involved in maintenance functions. Four cell division proteins (two contigs from the PCG versus POH comparison plus two contigs from the POL versus POH comparison) are involved in the mitotic cell cycle and mitotic spindle/sister chromatid organization. Immunity-related proteins include six C1q domain containing contigs (one contig each from the PFA versus PCG and PCG versus POL comparisons, and two contigs each from the PCG versus POH and POL versus POH comparisons) and four myticin c contigs (one from the PCG versus POH comparison and three from POL versus POH comparison). Lastly, proteins involved in ribosome formation and translation include three 60S ribosomal proteins (one from the PCG versus POH comparison and two from the POL versus POH comparison) and eight 40S ribosomal proteins (four from the PCG versus POH comparison and four from the POL versus POH comparison).

Patterns of functional enrichment were more complex for the much larger set of genes whose transcript levels changed significantly without a corresponding change in protein abundance. Only one unannotated gene met the criteria for the EFA versus PFA comparison, and no functionally enriched GO categories were identified for the PFA versus PCG comparison (184 genes). For the PCG versus POL comparison, the 146 genes in set 4 had overrepresentation of nine biological process GO terms related to regulation of biosynthetic and metabolic processes (Table S3). These genes with discordant transcript and protein expression patterns included four genes involved in the regulation of transcription by RNA polymerase II (histone H2A.V, high mobility group-T protein-like, NK-tumor recognition protein and heterogeneous nuclear ribonucleoprotein K-like isoform X1). All four of these transcription regulation genes were expressed significantly higher in the PCG group. For the PCG versus POH comparison, overrepresented biological process categories among the 512 genes in set 4 included protein folding (e.g. four peptidyl-prolyl cis-trans isomerases, 10 kDa mitochondrial HSP and prostaglandin E synthase 3), vesicle mediated transport (e.g. three coatomer subunits, ras-related protein Rab-1A and 2, and ADP-ribosylation factors 4 and GGA1) and unfolded protein binding [e.g. eight T-complex protein 1 subunit contigs (Fig. S2), endoplasmic reticulum chaperone BiP, and 10 HSPs]; all genes in these categories were expressed more highly in POH. Finally, for the POL versus POH comparison (607 genes), overrepresented GO categories included protein folding and unfolded protein binding [e.g. peptidyl-prolyl cis-trans isomerases B and FKBP4 isoform X1; endoplasmic reticulum chaperone BiP; T-complex protein 1 subunits gamma, alpha, beta, delta, epsilon, eta, theta, zeta (Fig. S2); and HSPs 10, 70, 75 and 90] and purine nucleotide and ribonucleotide binding (e.g. three stress granule-associated contigs including polyadenylate-binding protein 1 and 4; ras GTPase-activating protein-binding protein 2 isoform X1). Transcript expression for all these contigs was higher in the POH group.

### Genes stably expressed across microhabitats

A total of 541 genes out of the 1519 examined (35.6%) remained stably expressed at the transcript and protein level across all five treatments (i.e. no DE). This set of genes was not functionally enriched for any GO terms, but 34 genes were identified as structural constituents of the ribosome (e.g. 11 40S and 20 60S ribosomal proteins) and 20 were identified as integral components of the membrane (e.g. transport proteins such as ADP, ATP carrier protein 3, sodium/potassium-transporting ATPase subunits alpha and beta, and copper transport protein ATOX1-like and receptors such as mitochondrial import receptor subunit TOM70, neuronal acetylcholine receptor subunit alpha-2 isoform X1 and G-protein coupled receptor 158; Table S3).

### Co-expression networks

**Fig. 4.**
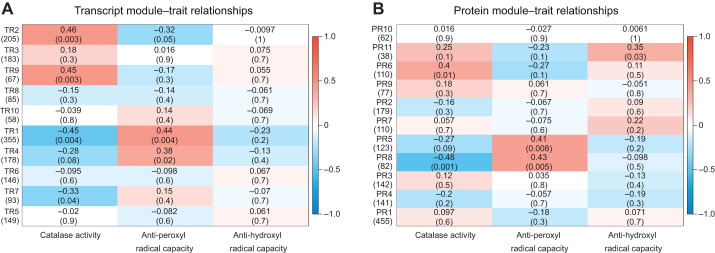
**Different expression modules identified across RNA transcript and protein expression data.** Each row corresponds to a module, with numbers in parentheses underneath each module label on the *y*-axis indicating the number of genes in that module. Modules are ranked by the number of genes they contain; for example, TR1 contains the highest number of transcripts, and TR10 contains the lowest number of transcripts. Each column represents a physiological trait quantified in gill tissue of the same individual mussels in Jimenez et al. (2015). Values in the top row of each square represent the transcript (A) or protein (B) correlation coefficient between each module eigengene and each physiological trait. Values in parentheses in the bottom row of each cell provide *P*-values. Colors represent the strength and direction of correlation, with red indicating a positive correlation and blue indicating a negative correlation.

Using the WGCNA algorithm, 10 and 11 unique co-expression modules were identified for transcript and protein expression, respectively. Interestingly, in both datasets, modules that were correlated with catalase activity tended to be correlated in the opposite direction with anti-peroxyl radical capacity (Fig. 4).

Two transcript modules (TR2 and TR9) were significantly positively correlated with catalase activity, while the TR1 and TR7 modules were negatively correlated with catalase. The TR2 module contains 205 genes, and average expression of genes in this module was highest in the PFA and EFA groups and lowest in the POH group. Contigs in the TR2 module had overrepresented GO terms related to the microtubule cytoskeleton, cilium assembly and movement (Table S4). There was a significant correlation between a gene's significance for catalase enzyme activity and its calculated membership in the TR2 module (*R*=0.7, *P*<2.2e-16). We identified 46 genes with high gene significance (>0.4) and high TR2 module membership (>0.8) (Table S4): six of these were dynein chains that are associated with microtubule motor activity and three were cilia- and flagella-associated proteins. The TR9 module contained 67 genes and was expressed highest on average in the POL group. There were no functionally enriched GO terms in this module. Gene significance for catalase enzyme activity was significantly correlated with calculated membership in the TR9 module (*R*=0.6, *P*=1e-07), and the TR9 module contained 13 contigs with high gene significance and high module membership. These central module contigs represented functions such as calcium ion binding (e.g. 1 annexin, 1 dystoni, and 1 EF-hand domain-containing contig; Table S4).

Two other transcript modules (TR1 and TR4) were significantly positively correlated with anti-peroxyl radical capacity. The TR1 module contained 355 genes, was functionally enriched for GO terms related to unfolded protein binding and protein folding, and had highest average expression in the POH treatment. There is a significant correlation between a gene's significance for anti-peroxyl radical capacity and its membership in the TR1 module (*R*=0.65, *P<*2.2e-16). Forty-eight genes in this module had high gene significance and high module membership, including HSP10, HSP30, HSP70, two dnaj homologs and two 78 kDa glucose-regulated proteins (Table S4). The TR4 module contained 178 genes and was functionally enriched for Ras protein signal transduction. This module had highest average expression in the PFA and POL groups, and lowest average expression in the POH group. A gene's calculated membership in the TR4 module was significantly correlated with its significance for anti-peroxyl radical capacity (*R*=0.64, *P<*2.2e-16). Twelve central module contigs with high gene significance and high module membership included membrane-related proteins such as vesicle-associated membrane protein-associated protein A, charged multivesicular body protein 4b, erlin-2 and Saposin-related isoform A (Table S4).

Although one or more protein modules were correlated with each of the physiological metrics, none of the genes in these protein modules had module memberships above 0.8 to meet criteria as important module nodes (Table S4). Specifically, genes in the PR6 module showed a significant correlation between module membership and significance for catalase enzyme activity (*R*=0.36, *P*=1.2e-4). Both the PR5 and PR8 modules had a significant correlation between module membership and gene significance for anti-peroxyl radical capacity (*R*=0.3, *P*=6.9e-4 and *R*=0.6, *P*=6.4e-3, respectively). Finally, the PR11 protein module was the only module across transcript and protein significantly positively correlated with anti-hydroxyl radical capacity, and it was functionally enriched for structural constituents of the ribosome and translation GO terms.

## DISCUSSION

We used a multi-omics approach, combining RNA-seq transcriptomics and proteomics, to investigate gene expression differences across ecologically relevant treatments in the intertidal mussel *M. californianus.* Our results clearly indicate plasticity in the molecular phenotype of adult mussels moved between environmental contexts. This plasticity was observed at the global level using PCA, within correlated modules of genes, and in differential expression of specific gene products. Both transcriptomic and proteomic data similarly differentiated our treatments, mirroring the separation of treatments observed in multivariate space for nine physiological metrics of antioxidant capacity from the same individuals (see Jimenez et al., 2015). However, the isolated datasets led to disparate conclusions regarding the underlying physiological mechanisms; we discuss several examples of such disparities below. Overall, both transcript- and protein-level regulation appear to play important, and non-overlapping, roles in microhabitat acclimatization of this species. Therefore, both types of regulatory processes must be considered when examining organismal responses to environmental stress and/or climate change.

### Acclimatization to stressful microhabitats involves constitutive transcript expression and post-transcriptional regulation of chaperones

Disparities between transcriptome and proteome were perhaps most pronounced in the protected outplant high treatment. Mussels sampled from the POH treatment had not yet experienced a prolonged period of emersion and associated thermal stress and valve closure for the day. Nevertheless, there were 25 contigs involved in the unfolded protein response that were significantly DE at the transcript, but not the protein, level in the PCG versus POH and POL versus POH comparisons. This includes molecular chaperones such as HSP30C, HSP70, HSP60, HSP70B2, HSP10, HSP70-4, dnaj subfamily A member 1s, T-complex protein 1 subunits beta, zeta, epsilon, alpha, delta and theta, and endoplasmic reticulum chaperone BiP. The T-complex protein 1 subunits act as molecular chaperones for cytoskeletal components such as actin and tubulin (Zhao et al., 2017), and the cytoskeleton has previously been proposed to be a weak link in heat tolerance of intertidal mussels (Lockwood et al., 2010). Similar patterns of unfolded protein-associated transcript expression have been observed previously in *M. californianus* individuals sampled from low and high intertidal sites throughout the tidal cycle (Gracey et al., 2008). Those authors hypothesized that such a pattern of gene expression indicated that mussels used a preparatory strategy between inevitable stressful low tide periods. The protein expression data added in the present study are consistent with this hypothesis, suggesting that increased expression of these proteins is only triggered by the physical denaturation of cytoskeletal proteins during stressful emersion conditions. Such an ‘on-demand’ mechanism could represent an energy-saving strategy, given the costs associated with chaperone translation and replacement (Dong et al., 2022). We further hypothesize that mussel acclimatization to stressful microhabitats such as the outplant high site involves storing stress-response transcripts in a translationally inactive, but highly stable form (Blevins et al., 2019; Liao et al., 2021). This hypothetical mechanism would enhance the preferential translation of stress-response genes often identified as part of the ‘integrative stress response’ (Zhao et al., 2002).

Interestingly, we found evidence consistent with a global suppression of constitutive translation rates at the outplant high site. Specifically, five contigs that are structural constituents of ribosomes (40S and 60S) are downregulated in POH relative to all other treatment groups. Although a decrease in ribosomal density could prevent the high number of stress-response transcripts from being translated before they are required (Blevins et al., 2019; Zarai et al., 2016), it is likely that decreased protein synthesis is an energetic compromise resulting from the reduced time for feeding (and reduced growth) at high-intertidal sites.

Overall, the pattern of constitutive transcript, but not protein, expression is consistent with a ‘threshold’ model for translational activation of the unfolded-protein response. Although we did not collect time-series data to explicitly evaluate this threshold, previous work indicates a field temperature of 28°C is not sufficient to trigger hsp70 protein expression in *M. californianus* (Roberts et al., 1997). Other species show similar patterns: in the mussel *M. galloprovincialis*, small HSPs that refold damaged cytoskeletal proteins do not become abundant until exposure to 32°C (Tomanek and Zuzow, 2010), and in zebrafish, transcription and translation of Hsp70s were only coupled at extreme heat stress temperatures (Mottola et al., 2020). Future studies could investigate the temperature thresholds and signaling mechanisms involved in triggering the translation of constitutively expressed molecular chaperone transcripts in *M. californianus*.

### Differential transcript expression related to ciliary activity and feeding

We hypothesize that patterns of differential expression of cilia and motility-related genes is related to differences in feeding time across treatments. Genes related to cilia and motility showed unique expression between field-acclimatized protected and outplant high groups in the transcript PCA, as well as differential transcript expression between the field-acclimatized protected group and the common garden group. These cilia-associated genes exhibited higher expression in the field-acclimatized treatment. *Mytilus californianus* is a filter feeder that relies on movement of particles through ciliary action (Bezares-Calderón et al., 2020). Thus, individuals at lower sites that are more frequently submerged can feed more than individuals at higher sites that are more frequently emersed. Feeding behavior is a large evolutionary driver in *Mytilus* species; ciliary structural maintenance genes are overrepresented in the *M. galloprovincialis* genome (Murgarella et al., 2016).

This feeding behavior hypothesis is supported by previous work in *Mytilus* species. *Mytilus californianus* individuals at low and wave-exposed sites grow 6- to 9-fold more than high-shore, wave-sheltered individuals (Connor and Robles, 2015). Furthermore, fast growth is associated with increased rates of feeding and overexpressed ciliary activity transcripts. Higher expression of dynein, a microtubule motor protein found in cilia, is correlated with higher filtering activity (Prieto et al., 2019). One seemingly contradictory finding is that individuals in the common garden treatment, which were constantly submerged, did not show high expression of these cilia-associated genes. However, our previous work has shown that growth is low under common garden conditions (Gleason et al., 2018), suggesting that feeding behavior may be lower than expected in this environment. Mussels in the lab common garden treatment were fed daily, but they may have experienced less total access to food than field counterparts.

Notably, the ciliary genes were not differentially expressed at the protein level in mussel gills. We speculate that elevated transcript levels support higher turnover of a structurally limited standing stock of cilia-associated proteins; only so many cilia can occupy the gill epithelial surface. Protein turnover in gill epithelial cilia of other bivalve mollusks such as the bay scallop *Aequipecten irradians* has been reported (Stephens, 1996), although to our knowledge, a positive correlation between ciliary activity and cilia protein deterioration/turnover has not been demonstrated. Protein lifetime estimates would be useful to test this conjecture.

### Differential transcript expression related to temperature extremes

The protected outplant high (POH) treatment group showed a clear separation from all other treatments in multivariate space, which is likely driven at least in part by the large differences in maximum temperatures between the POH and outplant low (POL) sites (Table 1; Jimenez et al., 2015). Moreover, when identifying DE transcripts in the POH compared with the POL and PCG groups, protein-folding genes were significantly overrepresented. Our results indicate that protein folding in different locations of the cell, and for proteins at different stages of synthesis, are important for survival in stressful microhabitats.

The expression data also indicate that HSPs of varying molecular weights [dnaj (HSP40), HSP70, HSP75 and HSP90] are expressed differently across microhabitats. Notably, different isoforms seem to be used in microhabitats that vary in the degree of temperature stress. For example, two different HSP90 and dnaj subfamily member B isoforms were significantly differentially expressed between the POL and the POH treatment groups. However, for both genes, one of these isoforms was more highly expressed in the POL group, and the other was more highly expressed in the POH group. Similar results have been found in other marine invertebrates such as sea anemones and *M. galloprovincialis*, in which different HSP contigs are upregulated under unique conditions (e.g. Hofmann, 1999; Snyder et al., 2001; Waller et al., 2018).

Our results also illustrate the importance of the endoplasmic reticulum (ER) chaperone system in coping with stressful microhabitats. This stress pathway performs quality control for the folding of newly synthesized proteins translocated into the ER (Adams et al., 2019). For example, calnexin, calreticulin and UDP-glucose:glycoprotein glucosyltransferase 1 all work together to ensure that glycosylated proteins fold properly (Hebert et al., 1995; Sousa and Parodi, 1995). High expression of calnexin and/or calreticulin transcripts under heat stress has also been observed in krill (Clark et al., 2011), scallops (Yang et al., 2021), corals (Maor-Landaw et al., 2014) and mussels (Negri et al., 2013).

### Transcriptome and proteome discrepancies inform future omics studies

In general, major disparities in the transcriptome and proteome were identified in these expression datasets, mirroring previous findings (Fessler et al., 2002; Huber et al., 2004; Jayapal et al., 2008; Tian et al., 2004). Although biological processes such as differential translation rates, degradation of mRNA in the cell, and distinct lifespans of proteins versus mRNA could be affecting these patterns, genes that are regulated in a corresponding direction at both transcript and protein levels are clearly the exception in these data, rather than the rule (Fig. 3). For example, except for the EFA versus PFA comparison, significantly more transcripts versus proteins were DE between treatments. Those genes DE at the transcript but not the protein level represent GO terms such as protein folding (the chaperones discussed above), vesicle mediated transport, and purine nucleotide and ribonucleotide binding. Although some contigs are DE at both the transcript and the protein levels, in 29% of those instances (concentrated in the POL versus POH comparison) the two levels are regulated in opposing directions. Furthermore, a subset of genes DE at the protein but not the transcript level represents distinct maintenance functions such as cell division, immunity and ribosome formation. The addition of proteomics data provides information about changes in these cellular and molecular functions that are not captured when examining transcript expression alone.

However, employing a proteomics approach does entail challenges. Proteomics methods are noisier than transcriptomic methods (Hurley et al., 2018; Crowell et al., 2019), and this higher background noise can limit the detection of DE proteins (De los Santos et al., 2021). Moreover, fewer genes are identified using proteomics versus RNA-seq, and more missing protein expression values must be imputed (Hall et al., 2020). In previous work, we hypothesized that our imputation method could lead to underestimates of protein expression variation within a treatment (Tanner et al., 2022). In this study, this artificial reduction in treatment variance could overestimate the number of DE proteins detected. However, this potential effect is not expected to change our conclusions that constitutive transcript, but not protein, expression of molecular chaperones is key for acclimatization to stressful microhabitats, or that inferring protein abundance from transcript levels can result in ‘false positives’ (see below). If anything, additional data would be likely to further reinforce these patterns.

Taken together with those caveats, the present results suggest the possibility of ‘false positives’ when attempting to infer from RNA-seq transcript expression levels to protein abundance, with a comparatively small set of ‘false negatives’. However, this ratio appears to be treatment dependent. For example, 348 unique transcripts were shared in set 4 (genes DE at the transcript but not protein level) between the comparisons involving the POH treatment; this list includes numerous HSPs and other genes involved in the cellular stress response. In contrast, only 13 unique proteins were DE without a corresponding change in transcript expression (set 3) in the same comparisons involving POH. Contrasting these numbers with those for the EFA versus PFA comparison (1 transcript in set 4; 29 proteins in set 3) clearly illustrates the shifting state of transcriptional and translational regulation across treatments. For example, the higher number of DE proteins versus transcripts in the EFA versus PFA comparison suggests that acclimatization to temperature variation caused by presence or absence of wave splash involves post-transcriptional mechanisms. Additional data are needed to clarify these patterns and to examine their generality across species and experimental conditions.

The stronger relationship between transcript and protein expression in the POL and POH treatments could be related to preferential translation of proteins needed under stressful conditions (Liu and Qian, 2014) or a high proportion of DE genes with AAG codons, which are translated efficiently during heat stress (Wu et al., 2021). Alternatively, the higher correlation in these treatments could be due to a higher proportion of DE transcripts (Koussounadis et al., 2015). Overall, our results match previous findings in wheat that post-transcriptional regulation affects protein expression more under severe versus mild stress conditions; transcriptional regulation explained only 6% of protein expression at 30°C, but 31% of protein expression at 40°C (Wu et al., 2021). This should be considered when planning future -omics studies. Particularly when the goal is to investigate responses to ‘typical’ conditions (i.e. no to mild stress) or to different locations (as in latitudinal comparisons), RNA-seq alone may not accurately predict which changes occur at the protein, and thus physiological, level.

### Co-expression modules also differ between the transcriptome and proteome

As for the univariate analyses, the WGCNA approach identified unique co-expression patterns within the transcriptomic and proteomic datasets. Overall, we identified transcript and protein co-expression modules that were correlated with physiological data from the same individuals. However, important disparities exist between the transcript and protein expression data. For example, the PR11 protein module that was significantly enriched for ribosome and translation GO terms was significantly correlated with anti-hydroxyl radical capacity, but no transcript modules were correlated with this physiological trait. In addition, two transcript modules (TR1 and TR4) and two protein modules (PR5 and PR8) were significantly positively correlated with anti-peroxyl radical capacity. The transcript modules are enriched for unfolded protein binding and Ras protein signal transduction, respectively; however, the protein modules that correlated with this same physiological metric are composed of fewer genes, have no enriched GO terms, and exhibit lower module membership values. Similar patterns emerge for transcript and protein module correlations with catalase enzyme activity. These results further demonstrate that the transcriptome and proteome datasets supplement each other and together identify candidate genes related to physiological metrics that would be missed if only one of the two datasets was used.

### Conclusions

This study suggests that transcriptional and protein-level regulation play unique roles in the acclimatization of the intertidal mussel *M. californianus* to distinct microhabitats. Specifically, acclimatization to the outplant intertidal sites involves a higher number of genes with strong correlation between transcript and protein expression, along with constitutive transcript (but not protein) expression of select molecular chaperones involved in the unfolded protein response. Variations in temperature and feeding time affect transcript expression patterns. Genes required for general maintenance functions such as immunity and cell division seem to be regulated at the protein level. Co-expression modules of transcript and protein are correlated with physiological metrics, but the co-expression structure differs markedly between transcriptome and proteome. Overall, our results provide further insight into how intertidal invertebrates survive in their extremely variable environment and illustrate the additional information gained by conducting studies assessing multiple levels of gene expression regulation.

## Supplementary Material

10.1242/jexbio.245962_sup1 Supplementary information

Table S1. The contig identity and Gene Ontology (GO) category or categories of all genes with a significant correlation between their normalized expression values and Dimension 1 or 2 of the transcript and protein PCAs in Fig. 1.

Table S2. Overrepresented Gene Ontology (GO) functions and corresponding normalized expression values in each treatment (average±b 1 SEM) for differentially expressed transcripts in the (i) PCG v. POL, (ii) PCG vs. POH, and (iii) POL vs. POH treatment comparisons. No overrepresented functions were identified for the EFA vs. PFA and PFA vs. PCG comparisons and thus no genes from those comparisons are presented here. The treatment group written first in each comparison is the reference, and asterisks on individual gene names indicate transcripts that showed significantly higher expression (indicated by FDR values from EdgeR) in the reference group.

Table S3. List of gene names and associated Gene Ontology (GO) terms for those contigs that were 1) differentially expressed (DE) at both the RNA and protein level and share the same direction of expression change (aka match); 2) those that are DE at both levels but change expression in opposite directions (aka mismatch); 3) those that are DE at the transcript but not the protein level; 4) those that are DE at the protein but not the transcript level; and 5) stably expressed at both the RNA and protein level (no DE) in each of the five treatment groups. The treatment group written first in each comparison is the reference. Upregulated = higher expression in the reference group; downregulated = lower expression in the reference group

Table S4. Contigs in WGCNA transcript and protein modules that are significantly correlated with catalase enzyme activity at the transcript (TR2 and TR9) or protein level (PR6), with anti-peroxyl radical capacity at the RNA transcript (TR1 and TR4) or protein level (PR5 and PR8), and with anti-hydroxyl radical capacity at the protein level (PR11). Annotation information, gene significance with each respective physiological metric, and module membership values are provided for each contig.
