# Supplementary material for: Multi-omics reveals largely distinct transcript- and protein-level responses to the environment in an intertidal mussel
Source: J Exp Biol. 2023 Nov 21;226(22):jeb245962. doi: 10.1242/jeb.245962 (PMC10690110; doi:10.1242/jeb.245962)
Supplement: Supplementary information [file jexbio-226-245962-s1.pdf]

A.

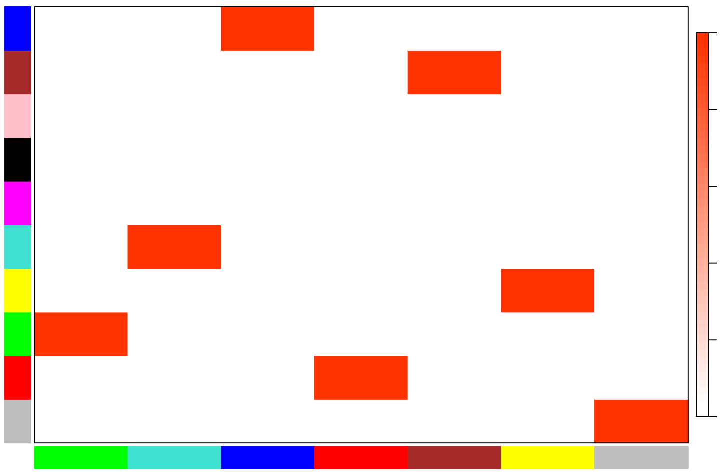

B.

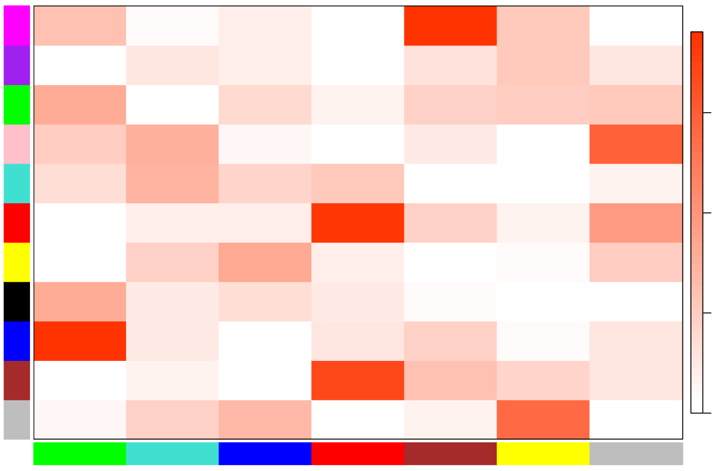

Fig. S1. Correspondence of consensus modules created from both transcript and protein expression data and A) transcript or B) protein-specific modules. Each row of the table represents one transcript or protein-specific module, and each column represents one consensus module. Numbers in each cell of the table represent the number of genes found in both respective intersecting modules, with a lighter coloring indicating a low level of overlap between the respective transcript or protein-specific and consensus modules. Unlike the transcript-specific modules, most protein-specific modules did not have a single corresponding consensus module counterpart, indicating that the consensus modules do not adequately represent the protein data.

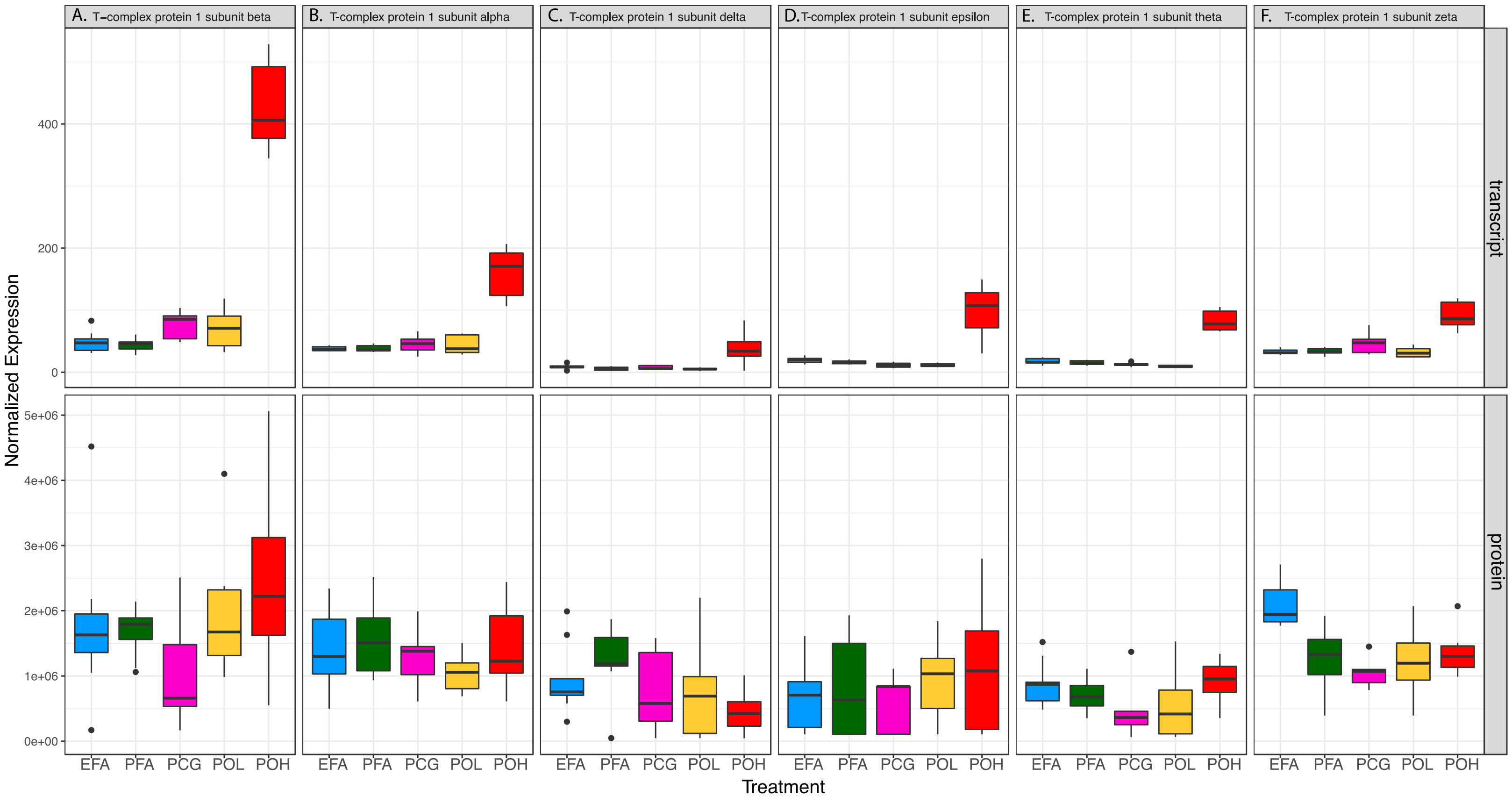

Fig. S2. Normalized transcript (top row) and protein (bottom row) expression values of T-complex protein 1 subunits beta (A), alpha (B), delta (C), epsilon (D), theta (E), and zeta (F) involved in the unfolded protein response that were significantly differentially expressed at the transcript, but not the protein, level in both the PCG vs. POH and POL vs. POH comparisons.

Table S1. The contig identity and Gene Ontology (GO) category or categories of all genes with a significant correlation between their normalized expression values and Dimension 1 or 2 of the transcript and protein PCAs in Fig. 1.

Available for download at
https://journals.biologists.com/jeb/article-lookup/doi/10.1242/jeb.245962#supplementary-data

Table S2. Overrepresented Gene Ontology (GO) functions and corresponding normalized expression values in each treatment (average ± 1 SEM) for differentially expressed transcripts in the (i) PCG v. POL, (ii) PCG vs. POH, and (iii) POL vs. POH treatment comparisons. No overrepresented functions were identified for the EFA vs. PFA and PFA vs. PCG comparisons and thus no genes from those comparisons are presented here. The treatment group written first in each comparison is the reference, and asterisks on individual gene names indicate transcripts that showed significantly higher expression (indicated by FDR values from EdgeR) in the reference group.

Available for download at
https://journals.biologists.com/jeb/article-lookup/doi/10.1242/jeb.245962#supplementary-data

Table S3. List of gene names and associated Gene Ontology (GO) terms for those contigs that were 1) differentially expressed (DE) at both the RNA and protein level and share the same direction of expression change (aka match); 2) those that are DE at both levels but change expression in opposite directions (aka mismatch); 3) those that are DE at the transcript but not the protein level; 4) those that are DE at the protein but not the transcript level; and 5) stably expressed at both the RNA and protein level (no DE) in each of the five treatment groups. The treatment group written first in each comparison is the reference. Upregulated = higher expression in the reference group; downregulated = lower expression in the reference group

Available for download at
https://journals.biologists.com/jeb/article-lookup/doi/10.1242/jeb.245962#supplementary-data

Table S4. Contigs in WGCNA transcript and protein modules that are significantly correlated with catalase enzyme activity at the transcript (TR2 and TR9) or protein level (PR6), with anti-peroxyl radical capacity at the RNA transcript (TR1 and TR4) or protein level (PR5 and PR8), and with anti-hydroxyl radical capacity at the protein level (PR11). Annotation information, gene significance with each respective physiological metric, and module membership values are provided for each contig.

Available for download at
https://journals.biologists.com/jeb/article-lookup/doi/10.1242/jeb.245962#supplementary-data

Journal of Experimental Biology • Supplementary information
